# Is a randomised controlled trial of take home naloxone distributed in emergency settings likely to be feasible and acceptable? Findings from a UK qualitative study exploring perspectives of people who use opioids and emergency services staff

**DOI:** 10.1186/s12873-024-00987-y

**Published:** 2024-04-29

**Authors:** FC Sampson, J Hughes, J Long, P Buykx, SW Goodacre, H Snooks, A Edwards, Bridie Evans, Jenna Jones, Chris Moore, Sasha Johnston

**Affiliations:** 1 Division of Population Health, University of Sheffield, Sheffield, UK; 2 School of Humanities, Creative Industries and Social Sciences, University of Newcastle, Newcastle, Australia; 3 Department of Medicine, Swansea University, Swansea, UK; 4 Division of Population Medicine, Cardiff University, Cardiff, UK; 5 Welsh Ambulance Service NHS Trust, Cardiff, UK; 6 South West Ambulance Service NHS Trust, Exeter, UK

**Keywords:** Take home naloxone, Drug overdose prevention, Qualitative research, Emergency services, Feasibility study, Patient perspectives

## Abstract

**Objective:**

Distribution of take-home naloxone (THN) by emergency services may increase access to THN and reduce deaths and morbidity from opioid overdose. As part of a feasibility study for a randomised controlled trial (RCT) of distribution of THN kits and education within ambulance services and Emergency Departments (EDs), we used qualitative methods to explore key stakeholders’ perceptions of feasibility and acceptability of delivering the trial.

**Methods:**

We undertook semi-structured interviews and focus groups with 26 people who use opioids and with 20 paramedics and ED staff from two intervention sites between 2019 and 2021. Interviews and focus groups were recorded, transcribed verbatim and analysed using Framework Analysis.

**Results:**

People using opioids reported high awareness of overdose management, including personal experience of THN use. Staff perceived emergency service provision of THN as a low-cost, low-risk intervention with potential to reduce mortality, morbidity and health service use. Staff understood the trial aims and considered it compatible with their work. All participants supported widening access to THN but reported limited trial recruitment opportunities partly due to difficulties in consenting patients during overdose. Procedural problems, restrictive recruitment protocols, limited staff buy-in and patients already owning THN limited trial recruitment. Determining trial effectiveness was challenging due to high levels of alternative community provision of THN.

**Conclusions:**

Distribution of THN in emergency settings was considered feasible and acceptable for stakeholders but an RCT to establish the effectiveness of THN delivery is unlikely to generate further useful evidence due to difficulties in recruiting patients and assessing benefits.

**Supplementary Information:**

The online version contains supplementary material available at 10.1186/s12873-024-00987-y.

## Background

Accidental overdose and death from opioids is a significant public health problem, with around 100,000 annual deaths globally, with relatively high rates particularly in high-income countries [1–4]. Non-fatal overdose is associated with long-term morbidity, and increased health service use [5, 6]. Rapid administration of the opioid antagonist, naloxone can reduce likelihood of respiratory arrest and death from opioid overdose [7]. In the UK, regulation of naloxone provision was changed in 2015 to enable supply of naloxone without prescription by people working for drug treatment services [8]. Take home naloxone (THN) distribution programmes supply THN to people at risk of overdose for swift administration by people without formal medical training, prior to contacting emergency services [9]. These programmes are usually managed within the community, mainly by specialist drug services or police departments [10, 11]. More recently, programmes have been developed to administer THN through emergency services, either through provision of THN kits or prescription of THN [12–16].

Despite significant evidence about the benefits of naloxone as an effective drug to reverse opioid overdose, evidence about the effectiveness of THN programmes in reducing morbidity and mortality from opioid overdose either in specialist drug settings or emergency service settings is limited [17]. Small observational studies have shown that distribution of THN in EDs is likely to be feasible, but uptake has been low, particularly when THN has been delivered as prescription rather than kit [16, 18–21] Although distribution via ambulance services may be more effective due to many opioid users’ reluctance to travel to ED, evidence of feasibility within this field is even more limited. Notably there is a lack of evidence around risks of inadequate response or return to state of overdose following lay administration of THN [22–24], although a recent study identified THN provision does not appear to lead to increased substance use [25].

This qualitative study was part of a wider study, examining the feasibility of carrying out a fully powered randomised controlled trial (RCT) of THN distribution in emergency settings, using anonymised linked data to capture outcomes [26]. We conducted a qualitative evaluation of the feasibility and acceptability of the trial with key stakeholders [27, 28] Specifically, we spoke to people who use opioids to understand their perspectives on the acceptability of THN delivered from emergency settings, and to emergency services staff involved in delivering the feasibility study to understand whether (a) a randomised controlled trial of THN would be feasible and (b) whether delivery of THN in emergency settings would be feasible.

## Methods

The trial recruited from two intervention and two matched control Emergency Departments and their associated Ambulance Services in England and Wales. The intervention is described within the protocol [26] and involved ED and ambulance staff providing education and distributing THN kits to patients consenting to receiving the kit within the TIME trial. Kits contained a multi-dose syringe of naloxone, intramuscular needles, and instructions for drug administration, basic life support and instructions for calling emergency services [28].

## Study design and setting

We used qualitative methods using semi-structured interviews and focus groups with two groups of key stakeholders: ED and ambulance staff from both study areas and people with experience of opioid use accessing treatment centres or third sector (charity-funded) support groups. The latter group were people with experience of injecting opioids but not directly involved in the trial. This was in response to Public and Patient Involvement (PPI) panel members and funder advice suggested recruitment of trial participants would not be feasible. We therefore used this group to represent potential recipients of THN.

## Selection of participants

People with opioid injection experience were recruited from two substance use treatment centres and one third sector support organisation within the two study areas. Potential participants for opioid user interviews (> 18, experience of injecting drug use or carer/partner of service user) were identified by staff within the drug treatment centres/third sector organisation.

We recruited staff from the 2 EDs and Ambulance Services in the intervention arm of the trial. We sampled purposively for organisation, sex and role. We initially planned to undertake staff baseline focus groups at 0–3 months, then at 6 months to understand how the intervention had been adopted. However, due to recruitment pauses in the trial, limited staff availability for focus groups and the impact of the COVID-19 pandemic, we adapted to conduct individual telephone semi-structured interviews while the trial was running. Participating staff were invited to take part in the interviews by the ED and Ambulance Service research leads. In total, 278 staff were trained to supply THN kits to eligible patients (132 ED, 146 ambulance), 60 THN kits were supplied to eligible patients as part of the trial (ED 52, ambulance 8) and eligible patients were not offered THN kits 164 times (ED 159, ambulance 5).

All participants were allowed to withdraw up to 4 weeks after the interview/focus group.

## Data collection

Topic guides were developed for the study (see supplementary file).

Staff topic guides were guided by Normalisation Process Theory (NPT) [28, 29] using the constructs of coherence (what the intervention involves & its purpose), cognitive participation (who has a role in delivering the intervention), collective action (how the intervention was delivered and what enabled or hindered uptake) and reflexive monitoring (assessment of effectiveness). NPT is suitable for use in feasibility studies, can be applied flexibly and can be used to help understand what people do rather than what they say they will do. Topic guides aimed to explore whether, why, and how THN did or did not become part of routine practice in each site during the feasibility trial. A focus group plus three individual interviews took place within a meeting room of one organisation, with remaining interviews undertaken by telephone.

Opioid user semi-structured interview topic guides focused on personal and peer group experiences of opioid use, overdose and their perspectives on the potential barriers and enablers to THN delivery by ambulance and ED staff. Interviews were undertaken face-to-face with participants within the recruiting organisations. All participants were offered a £20 Love2Shop voucher.

## Analysis

All interviews were recorded and transcribed verbatim. Data were organised using NVivo 12 ™. Due to the different focus of the two groups of stakeholders (one had experience in delivering the intervention, the other comprised potential recipients of the intervention), we analysed data sets individually then triangulated findings to identify areas of convergence and conflict. We analysed interviews using Framework Analysis [30], based broadly on the constructs of NPT (using the framework described in Huddleston et al. [31]), adding further themes relating to the trial itself rather than the intervention. Initial coding was undertaken by JH & JL (opioid users), JL and FS (staff) and a subset were double coded. The coding structure was further refined, and analysis undertaken after discussion between JH, FS, JL and PB.

## Patient and public involvement (PPI)

We involved PPI representatives from an established PPI addiction group (Sheffield Addiction Recovery Research panel). One PPI member was recruited to the study panel during the trial design phase but left for personal reasons during the recruitment phase. We attempted to recruit further PPI members to the study panel but were unable to secure a named individual so instead although we accessed expert members of the PPI group’s resources to review interview schedules and support the study throughout.

## Participants

In total we conducted one initial focus group with 8 ED staff and 12 semi-structured telephone interviews (8 paramedics, 4 ED staff) across 1 ED and 2 ambulance services, and 26 service users across three different organisations (one within ambulance service area 1 and two within ambulance service area 2). We were unable to recruit any ED staff at the ED in ambulance service area 2. Service users were either current or former opioid users, and one was a carer with past experience of opioid use. Staff interviews lasted an average of 46 min (range 27–57 min). Service user interviews lasted an average of 17 min (range 9–32 min). Characteristics of participants are described in Tables 1 and 2.

**Table 1:** Characteristics of participants: staff interviews and focus groups

Site	Data collection method	Data collection period	Gender	Role
ED1	Focus group	Aug 2019	Not recorded	1 ED doctor, 1 ED nurse, 1 paramedic, 1 paramedic working in ED. 4 ED staff, role not recorded
ED1	Interviews (face to face)	Aug 2019, Feb 2021	3 F, 1 M	1 ED doctor, 2 ED nurse, 1 paramedic
Ambulance 1	Interview (telephone)	Feb/Mar 2021	2 M, 1 F	3 paramedics
Ambulance 2	Interview (telephone)	Feb/Mar 2021	4 M	4 paramedics, 1 ED nurse/paramedic joint role.

**Table 2:** Characteristics of participants: service users

Area and organisation	Data collection method	Data collection period	Gender	Site ID
Ambulance 2 (Outpatient treatment service, city 1)	Interview (face to face)	May/June 2019	7 M, 1 F	1 (IDs 1–8)
Ambulance 2 (Outpatient treatment service, city 2)	Interview (face to face)	May/June 2019	7 M, 3 F	2 (IDs 9–18)
Ambulance 1 Third sector drug organisation	Interview (face to face)	May/August 2019	8 Gender not recorded	3 (IDs 19–26)

### Findings

We identified six main themes that explain whether a full RCT of THN distribution via emergency settings is likely to be feasible and acceptable.

1. **Staff and service users perceived delivery of THN in emergency settings to be appropriate and identified multiple benefits to expanding the availability of THN.**

Staff and service users understood the aims of the trial and how it related to their work practices/lives and were broadly supportive of the intervention as a low-risk, easy to use intervention. Participants perceived multiple potential benefits to the intervention in terms of reducing mortality and morbidity, but also wider benefits to the health service such as reduced ED attendance and ambulance journeys for future overdose.

If it will free up ambulance resources for genuine people. (Service user 8, site 1)

*And it is – I think it would be beneficial for all of us. I think we all would collectively agree that, you know, if we could get more people to sort of take it up, then it would hopefully, certainly reduce the effort that we have to go through sometimes to resuscitate these people. (ED nurse Ambulance 2)*

*The reduction of fatal overdoses, to us, makes it worthwhile straight away [Paramedic, ED1 focus group]*.

*Overall it would benefit all round because it would prevent admissions to A&E so I think it would be cost effective, and it’s a relatively simple thing to take on board and to cascade down to patients. [ED nurse/paramedic, Ambulance Service 2]*

Service users reported significant experience of overdose (experienced personally or witnessed) and high levels of awareness and understanding of overdose management (e.g. first aid, administering CPR), including the role of THN in overdose reversal. They described personal experiences of either giving somebody else THN, witnessing its use or having received it themselves.

*“I was lucky, yeah. It wasn’t actually on these premises [treatment centre] but it wasn’t far away. And there was a member of staff who had Naloxone in her bag and she brought me round”. [Service User 4, Site 3]*

*“I was on the stairwell.and I had my Naloxone with me and my friend went over and I phoned the ambulance. They asked me if I had one [THN kit] with me. I said I did and they told me to use it and I did”. [Service User 2, Site 3]*

Witnessing the impact of naloxone in opioid overdose reversal helped both staff and service users understand the benefits and impact of THN as an intervention (HU01, staff), with paramedics describing how attending overdoses where THN had been delivered made their job easier. Service users perceived overdose as a recognised but unacceptable side-effect of opioid use and highly valued naloxone as an intervention that could save lives. Both sets of stakeholders strongly welcomed the opportunity to widen access to THN and reduce potential harms associated with overdose.

*“Because I knew that it saved my mate’s life before I’m more than willing to have this on me”. (Service user 1, Site 2)*

*“Like I said, I mean, we all know that people shouldn’t be taking opiates and things like that, but they will. And if we can get it so we’re – we can stop some preventable deaths, a lot of people die from taking drugs. If we can help support them to stop them dying, and even if it gives them an education enough to get off the drugs in the end, you know, we can make it a little bit safer for them and give them that opportunity to actually get through it”. [Paramedic, Ambulance 2]*

2) **THN offered opportunities for empowering a population who were seen as having low levels of self-efficacy.**

Participants perceived the intervention to have potential to increase self-efficacy and empower both service users and their peer and support groups. Service users described how having a THN kit made them feel more secure and confident in dealing with friends or family who used opioids, providing a more immediate response than an ambulance. Staff described how the provision of THN gave them a positive sense of being able to help a population for whom they felt they could offer little support.

*I kind of felt – with the Naloxone there, I kind of felt in control. If she’d – if I’d given her the Naloxone and it hadn’t brought her round, the first thing I’d have done was ring an ambulance”. [Service User 5, Site 2]*

*So, that’s what I saw as the key bonus to this scheme, was that it wasn’t just about supporting and educating people and saying, you know, “Recognise this risk and call help”, but also being able to say, “This is something you can do”, and kind of like empowering them at the point of care. [Paramedic, Ambulance 1]*

*It is a nice thing to leave people with, it sort of shows that we care. It’s another thing we can say in good faith, we saw this person, we discharged them after a heroin abuse episode that went a bit awry and we have left them with a life saving drug. And I think that’s a nice thing to do to people. [ED doctor, ED1 interview]*

Both ED staff and paramedics viewed the delivery of THN as compatible with their job role. Paramedics particularly welcomed the opportunity to expand their scope of practice and increase the value of their interactions with patients. Some ED and paramedic staff viewed the interaction when delivering THN training as an opportunity for them to encourage users to engage in further treatment and health promotion conversations with a hard-to-reach population who may otherwise have little contact with health services.

*Even if 10 people refuse it, if one takes it and it helps them then that is a good thing*.

*From a point of caring for the patient, I don’t think there is a lot of difference really. It’s more than just offering them the chance to take the kit home. I guess that involves a bit more time and education, because we wouldn’t necessarily routinely go through all the recovery position and talking through effects of overdose and things before. [Paramedic, ED1 interview]*

*I think it enables practitioners to have health promotion conversations with people in that lifestyle. And really to some extent it’s a kind of make every conversation – make every contact count message but the patient goes away with something useful at the end of it.[ED doctor, site 1 interview]*.

3) **Participants supported widening access to THN but had concerns about limited opportunities for engaging patients via emergency services.**

Staff and users of opioids supported widening access to THN, although staff had some concerns about the potential to deliver significant increases in access through emergency settings. Whilst delivering THN in emergency settings offered opportunities to access populations who might not otherwise obtain THN (e.g. those not accessing community drug services etc.) there were concerns that patients coming out of overdose were not necessarily receptive to discussing how to use THN at this stage, often being confused or aggressive.

*Yeah. A lot of the time it’s those patients who then become quite aggressive, very sort of disrespectful. And when someone’s shouting and screaming at you, it’s very hard to – (Role not reported, Focus group ED1)*.

*And often, if we give them such you know, a rapid reversal of the opioid, some of them can become quite aggressive, because they come around, they don’t know what’s happened. They’re confused, they’re hypoxic, you know, they can become quite aggressive, and there’s not much room to work in the back of an ambulance sometimes. [Paramedic/ED nurse, Ambulance 2]*

Those using opioids acknowledged difficulties in engaging with healthcare professionals at this stage due to physical symptoms associated with overdose reversal, and immediate concerns about obtaining further opioids to combat these feelings. However, they recognised this as momentary and a necessary repercussion, given the alternative.

*I ended up giving her a second dose and she came round, and she was a bit ratty with me and, you know, I was glad I did it, really. Afterwards, not that day, but afterwards, she agreed that I’d done the right thing. (Service User 5, Site 2)*

*I think I’d still – knowing that, still use it [THN] because I think it’s still more important to have someone confront you than potentially lose a life, so yeah, I’d still administer it knowing that. (Service User 9, Site 2)*

Any person, I’d rather them be violent than die. (Service user 4, site 1)

Some service users reported reluctance to travel in an ambulance, attend the ED or even contact emergency services, due to concerns about being in possession of drugs when police were present, or unpleasant withdrawal effects following Naloxone administration. They recognised the potentially beneficial role for THN distribution by ambulance services but did not reflect on how the ED might usefully provide THN, lacking experience of ED attendance post overdose. Staff perceived opportunities for ambulance staff to deliver THN to be potentially greater than for ED staff, partly due to higher contact rates, but also due to having more contact within the community and opportunities to engage with friends and family.

*The thing is there’s a fear round drug users that if you start ringing 999, the police are going to come, but a life’s a life, isn’t it?” (Service User 2, site 2)*.

I *would say that a lot of times we would go to overdoses, that would then refuse to come in because you’d give them the NARCAN and they’d wake up, and have capacity to refuse. So it would, potentially, be good because you’re also in the environment of the house that they live in, with other people there in their actual environment. So I feel like I probably would have had more opportunities. [ED Doctor, site 1 Focus Group]*

*So, I think, obviously, the people that generally present to ED having overdosed, will be drowsy, you know, take a while to recover, which obviously means that they’re not going to be able to take information in well, or engage. And, as I’ve said before, at the point that they then become more awake, then they don’t always want to stay to engage. So, that would be a barrier, I think [Paramedic, ED 1 interview]*.

4) **Procedural problems and high levels of patient refusal contributed to low trial recruitment.**

The trial struggled to recruit staff who could recruit patients, and patients themselves, principally due to procedural problems relating to the timing and processes of the trial. Whilst the intervention was described as well resourced, with easy-to-follow training, engagement of staff differed between the two EDs. One ED recruited few staff and did not engage with the qualitative work. The other ED had a proactive research lead who championed the trial, which resulted in greater staff engagement and willingness counter problems in recruiting patients (e.g. engaging known opioid users attending ED).

High staff turnover in both ambulance services and EDs resulted in difficulties recruiting and training enough staff for the trial to be adequately cascaded and maintained. This was exacerbated by an extended recruitment period due to the COVID-19 pandemic which caused the trial to be paused, combined with a reported reduction in number of ambulance calls to overdoses during the national lockdown. Recruitment pauses early in the trial due to protocol changes also meant staff were unaware when the trial was reopened to recruitment so potential patients were missed.

*The difficulty was the trial had been running for quite a long time and the department has a really high turnover of staff. So I would train people and then they would leave, or there will be people that haven’t been trained in it that because they’re just coming through constantly, I couldn’t keep up. Also, even if they did have the training the trial’s been running over a period of two years so they might forget everything I’ve said or some of the things I’ve said.” [ED nurse, ED1 interview]*.

*I think it can be difficult from a medical staff point of view, because in A&E we have a fairly high turnover. So, a lot of SHOs are only in the department for four months. And I think there was a big push with the trial and, you know, putting up the posters and really trying to make people aware. But with COVID and obviously other things that have gone on, I don’t know that the message has been as clear or consistent throughout the whole time. [Paramedic, ED1]*

Recruitment protocols specified patients could be recruited if fully conscious. However, ambulance crews reported that management protocols for opioid overdose recommended keeping patients from fully coming out of overdose so that they were safe but not fully alert, which meant they were unable to recruit and consent them. Staff also reported high levels of patient refusal, partly due to difficulties in engaging during overdose, but also due to patients stating they already owned a THN kit that had been issued in the community.

*So, I know when I went through my paramedic training, there was a big emphasis on if the, you know, respiratory rate is good and the observations are good, just keep them in that groggy state until we can get to somewhere that’s more – that’s safer. Be that a hospital or wherever it might be. But yeah, it’s – I think a majority of colleagues that I’ve worked with have found the same problem. [Paramedic/ED nurse, Ambulance 2]*

5) **Limited wider commitment and other competing priorities influenced recruitment.**

Staff recruitment to the trial was limited. Participants indicated that the beliefs and behaviours required to enable widespread acceptance of the intervention may not have been held by all colleagues, with some evidence of wider scepticism about the provision of THN by emergency services. Concerns arose from doubts about the ability to make a significant difference at a population level and about the ‘safety net’ effect, suspecting opioid users might take a higher dose, when knowing Naloxone is on hand should they overdose. Some service users acknowledged this risk, but also reported opioid use was dictated by affordability.

*“You can only take what you can afford at the end of the day”. [Service user 8, Site 1]*

*“I don’t personally agree with them, but other paramedics on station I spoke to were like, “Why on earth are we doing this? This isn’t something we should be doing. We’re just giving them kits and we’re essentially encouraging them to overdose again.” Again, I disagree with that strongly, but I would say it’s split opinion a bit.” [Paramedic, Ambulance 2]*.

*“When we discussed it within our team, so over a cup of tea, I think there was a variation in enthusiasm for it. Some people wondering how effective it would be, feeling that it was difficult to target the people who would be most vulnerable. […] Some people felt it was, you know, a thimble full effect in a bucket full of problem”. [Paramedic, Ambulance 1]*

Other priorities during the period also detracted from the trial. Notably, the impact of the COVID-19 pandemic on emergency services staff left them with limited energy for what was perceived as ‘additional’ work. The timing of the TIME trial also coincided with a number of competing trials whose topics may have been perceived to be more appealing.

*We’re talking about staff groups who are traumatised and exhausted after a year of a lot going on. Um, and perhaps it’s just not been the optimal time to try and recruit people. [ED doctor, ED1 interview]*

*We also had quite a lot of other trials going on at the same time. So, I think you need to – if you’ve got lots of people involved in trials, sometimes they just tend to concentrate on one. Whereas we had quite a lot of trials going on at the same time as this one”. [Paramedic, Ambulance 1]*

6) **Distribution of THN by emergency services should be enabled but benefits are unlikely to be measurable within a larger RCT.**

Staff did not perceive widespread benefits to undertaking a full trial, due to difficulties in recruiting patients, as well as difficulties assessing the outcomes of the trial. The proliferation of THN kits distributed in the community by local services also meant that it would be difficult to attribute any change in outcomes to the distribution of kits by emergency services. Service users similarly perceived other channels (e.g. pharmacy) to be more appropriate for receiving THN.

*So my experience in [City] is I’d say – I understand the – I think there’s saturation – not saturation but I think that drug services have got a longer term relationship with people, have done very well. I don’t know, and having said I don’t know I’m not saying this in a cynical way, I don’t know how much of an impact us carrying give away Naloxone has been for patients, if you see what I’m saying.[ED doctor, ED 1]*.

*I think the chemist [good place to get THN] ‘cos where are they going to get their pins [needles] from. [Service User 5, site 1]*

However, both staff and people using opioids welcomed the incorporation of THN into their everyday practice, particularly if this could be expanded to incorporate friends and family into the distribution and other ambulance clinicians (e.g. ambulance technicians) being trained to provide the kits. Expanding the distribution of THN by emergency services was perceived as a low cost, low-risk intervention that may be highly beneficial to a small subset of opioid users who would not otherwise access THN (e.g., those not in contact with community drug services).

*I think it’s totally compatible with normal ED, a specific group of patients, definitely. […] The more normal it comes, the quicker you can do it. So like any intervention that comes in and is new, it’s difficult at first but then it gets easier as time goes on. [ED nurse, ED1 interview]*

*I suppose it’s just mindsets, basically. Because a lot of the things that come in, say, thromboprophylaxis for people who can’t have a weight-bearing cast. That was brought in, this is now the policy, this is the checklist. And then it’s just gradually instilled in people. So just part of the process. So I suppose it’s just around that, isn’t it, really, just getting it into people’s mindsets that if you see somebody who’s had an opioid overdose, that is just part of their patient journey, it’s supposed to be part of their assessment and treatment, two parts. [ED doctor, ED 1 focus group]*

*Yeah, I mean, I would like it to be rolled out as a standard operating procedure, as a care pathway that is available to all staff in [the ambulance service]. And you know, every ambulance carries a drug box with naloxone. And I would like us to be able and empowered to hand that out in an appropriate way, as part of a standard operating procedure. [Paramedic, Ambulance 1 interview]*

## Discussion

Stakeholders recognised the potential benefits of the intervention and supported it in principle, although there were problems with recruitment and delivery of the intervention within the trial. Stakeholders welcomed the opportunity to increase access to THN via emergency settings and the intervention was seen as simple, low risk and feasible, with potentially more support for distribution via the ambulance service. Difficulties associated with the trial delivery itself led to low patient recruitment and recognition that the impact of emergency service delivery of THN may be limited. Assessment of effectiveness was problematic due to significant availability of THN from other sources. There was recognition that wider engagement of emergency service staff to deliver the intervention may be limited, with emergency services staff at times struggling to engage patients, particularly given the current context of rising demand for emergency care services. However, staff who were engaged in the study felt that the potentially high level of benefit for a small population of patients to be worthwhile.

Service users recognised the difficulties associated with engaging themselves and peers during overdose situations, but similarly strongly supported the potentially life-saving intervention. Hawk et al. identified that opioid users wanted EDs to offer harm reduction resources such as THN and overdose prevention education [32]. Other studies have also supported the finding that staff are supportive of Emergency Service distribution of THN in principle. Sokol et al. reported provider attitudes showed increasing understanding towards opioid users, and a desire to feel that they are making a difference to patients’ lives [33]. Hawk et al. (2022) reported improving attitudes amongst ED staff towards opioid overdose survivors and a survey of staff in Canada and Australia showed around 9/10 of staff supported provision of THN in the ED [15, 18])

However, despite support in principle, studies found that clinicians were missing critical opportunities to prescribe naloxone in pilot THN programmes within the ED, with between 8 and 11% of eligible patients receiving a naloxone prescription [16, 19] and only around one in only ten prescriptions being converted when THN was prescribed rather than provided. Holland et al. interviewed pharmacists and ED physicians about theoretical provision of THN in the ED and identified similar willingness to provide THN, awareness of the potential benefits but also concerns of some negative attitudes from a minority of staff [34]. Chua et al. [16] highlighted prescription rates were four times higher for epinephrine after ED visits for anaphylaxis than for naloxone or buprenorphine for opioid overdose. This suggests that low prescribing in this population is due to factors relating to the condition being prescribed for and may result from stigma relating to this patient population. This supports our finding that although emergency service clinicians were supportive in principle, this may not translate into practice in part due to negative attitudes towards the population group being targeted.

Lebin et al. sought to identify predictors of receiving a naloxone prescription through their ED programme [19]. They found that users were less likely to be given a prescription if they had a history of opioid use in the past which suggests that many may already have had THN from another source at ED visit. Papp et al. were unable to detect a significant difference in patient outcomes for patients who received THN following ED attendance for overdose but the study was under-powered [13]. They similarly observed the likelihood that THN would be used by other patients than the people who were offered the THN.

A recent systematic review of opioid overdose interventions delivered in the ED suggested that THN distribution via emergency services is feasible and acceptable to patients but also highlighted the paucity of evidence of effectiveness of delivery in this setting [35] with systematic evaluation of outcomes unlikely. The authors identified that adequate staffing and role responsibility was required for sustainable implementation of any overdose prevention interventions.

## Limitations

This study was subject to a number of limitations. Part of the study took place during the COVID-19 pandemic which affected both the trial itself and recruitment into this qualitative study. The move from focus groups to individual interviews may have enabled staff to talk more openly but also meant we were unable to draw on interaction between team members to explore shared and divergent experiences of the implementation of the study. Participants who engaged with the study were likely to be more receptive to the delivery of THN in emergency settings than other staff who may not wish to engage in research, or who did not engage with the intervention, as suggested by the findings. We may have achieved a more representative sample if we had been able to recruit staff directly, rather than through research leads.

Due to anticipated problems in recruiting patients who received THN from Emergency Services we recruited service users who had experience of overdose and opioid use rather than patients who were offered the THN within this study. This means the user perspective could only explore opioid overdose and THN experiences and opinions outside the RCT. The sample were also people who were engaging with treatment programmes, who may be more responsive to carrying THN people who use opioids but were not engaged with treatment programmes.

Interviews with opioid users were conducted within the service treatment centres and participants were sometimes distracted, often providing short responses. Despite good initial PPI involvement, changes in personal circumstances of two PPI members meant they withdrew and it was then difficult to maintain a stable PPI group to inform our study interpretation phase. We were however able to draw on the Sheffield Addiction Recovery Research Panel (ShARRP) who regularly commented on study progress.

The study took place in the UK where THN is funded via local authority commissioning which may limit some of the transferability of findings, particularly given barriers to THN arising from lack of central funding [21]. However, cost was not highlighted as a significant barrier to implementation of THN programmes in a review incorporating majority US-based studies, with attidudinal barriers being highlighted as more important [36]. 

## Implications

Distribution of THN in emergency settings appears to be feasible and acceptable for all stakeholders and may widen access to THN for those not engaging with wider community drug services. Adoption of THN distribution within emergency settings appears to be a low-risk intervention and may offer benefit if normalised into everyday practice. Given the difficulties in maintaining recruitment of staff and ambulance or ED patients during the feasibility study, it appears unlikely that a full trial would be feasible. This demonstrates the importance of undertaking feasibility or pilot studies prior to undertaking full RCTs.

Further RCTs of THN in emergency settings may be limited by difficulties in recruitment, particularly where THN provision from other settings is widespread. Other observational evaluation methods to understand the effectiveness of THN provision may be required, following widespread distribution of THN kits and monitoring of drug-related deaths by interrupted time series methods. Extending THN provision to peers and family of people at risk of overdose could offer additional opportunities to improve outcomes from opioid overdose. Whilst emergency services staff recognised the benefit of THN as an intervention, there is some evidence that negative attitudes from some staff need to be overcome to maximise the potential for delivering THN in emergency settings. These attitudes need to be addressed, but also mean that provision of THN via other routes such as specialist drug clinics will still be paramount.

### Electronic supplementary material

Below is the link to the electronic supplementary material.

Supplementary Material 1

Supplementary Material 2

## Data Availability

The full dataset will not be shared. This study reports qualitative data and data sharing risks confidentiality and anonymity of the participants. Permission was granted for sharing anonymised quotations but not the full dataset. For further details please contact Fiona Sampson f.c.sampson@sheffield.ac.uk.
