# Supplementary material for: Is a randomised controlled trial of take home naloxone distributed in emergency settings likely to be feasible and acceptable? Findings from a UK qualitative study exploring perspectives of people who use opioids and emergency services staff
Source: BMC Emerg Med. 2024 Apr 29;24:75. doi: 10.1186/s12873-024-00987-y (PMC11057101; doi:10.1186/s12873-024-00987-y)
Supplement: Supplementary file 1 — Supplementary Material 1 [file 12873_2024_987_MOESM1_ESM.docx]

**Topic guide - HCP interviews or focus groups - 0-3 months**

Role, organisation, how long been in role (and how long worked in organisation/ as paramedic/dr/nurse etc.

How many people have you given THN to? (either personally or been with team when they have).

**Establishing the conversation:**

Thank you for coming today. The purpose of this interview/focus group is to talk about the trial which is being run at [Site name] regarding Take Home Naloxone and for which ambulance and ED clinical staff have been trained. So you know what is going to happen and to help us structure our conversation, I’m going to ask for your thoughts on four main topic *areas (topic areas to be displayed on flip chart/handout)*:

1. Firstly, we’ll talk about what you think the intervention involves and what its **purpose** is *(note: this relates to the Normalisation Process Theory [NPT] component of ‘coherence’)*
2. Then we’ll discuss **who** does or could have a role in delivering the intervention *(note: NPT component of ‘cognitive participation’)*
3. Then we’ll cover **how** the intervention is being delivered here and what would **enable or hinder its uptake** *(note: NPT component of ‘collective action’)*
4. Finally, we’ll talk about how you and your colleagues might **assess** the effects of delivering the intervention *(note: NPT component of ‘reflexive monitoring’)*

Some of these areas may overlap, so it’s OK if we end up covering these things in a different order. Is that all ok?

*At the end, I’ll also ask those of you who have been directly involved in delivering the intervention to patients or their families to complete a short survey about how many times you have delivered the intervention and much time you’ve spent on this activity.*

Our initial aim was to speak to people at the beginning of the trial, then 6 months in, but due to various circumstances, this hasn’t been possible. Instead we will ask you about how it is going now and ask you to reflect on how your understand of the trial might have changed since the outset.

**Topics:**

- 1. What the intervention involves and its purpose

To begin, imagine you/your staff are caring for someone who has overdosed on opioids. What does this intervention require you/them to do that is different from what you/they would have done before the TIME trial was in operation?

*Listen whether identify all components of intervention: (a) train patient in how to administer THN, (b) advise regarding aftercare and (c) training in resus. Prompt if not mentioned*

*Listen whether spontaneously expand to include all target groups: (a) those who OD, (b) those at risk of OD, (c) family/ friends. Prompt if not mentioned*

*Do you feel that other colleagues share your understanding of the intervention?*

What do you see as the **aim or purpose** of doing this (i.e. delivering the THN intervention)?

*Listen for potential benefits (e.g. save lives) (What are the benefits of the intervention?)*

*Listen for potential beneficiaries (e.g. wider social/family group, different risk groups, emergency services) (who do you think benefits from the intervention?)*

*Listen for ‘rejections’ of interventions aims (e.g. belief THN may encourage risk taking) (Do you think there may be any risks associated with the intervention?)*

*(If not yet emerged)* What do you see as the aim of delivering THN in an emergency care setting compared to other settings?

*Listen for comments on who the patient group is and their level of need – is this intervention relevant to few or many patients? Prompt if not mentioned*

6 MONTHS

Has your understanding of the aim and purpose of the intervention changed since you were first involved in the trial? What about other colleagues?

*Briefly summarise what was said about the intervention last time, including any uncertainties that were raised about the purpose or components of the intervention, perceived benefits/concerns about THN, etc*

- Is this still an accurate picture of how you see the intervention? Has there been any change in your understanding of the THN intervention since then? *(If yes, ask to expand)*
- What about other people you work with? Do you think there is a shared understanding? *(Ask to expand)*

The last time we were here, we also asked people to specifically reflect on the value of delivering THN in an emergency care setting compared to other settings (e.g. drug services). They tended to say…

*Summarise previous discussion about preferable settings*

- What would you say about this now?
- 2. Whose role is it to deliver the intervention?

In this trial, THN training is being provided to ambulance paramedics, doctors, and nurses.

- In your view, how compatible is delivering this intervention with your job role? With your colleagues’ roles? (do you see this as fitting in with your main job?)
- How compatible is the intervention with the overall purpose and work practices of this organisation? (does this intervention belong in the ED/ambulance service? Does the delivery of the intervention fit in with other work? Do you feel that other colleagues feel the same?)

*Listen for likely THN champions and possible gaps in delivery*

*Listen for examples of perceived synergy (e.g. this will ‘add’ to our response) or misalignment (e.g. organisational focus on emergency response, not prevention)*

*6 MONTHS*

Thinking back to when you were first involved in the trial, have there been any changes in who delivers the intervention, or is involved in the intervention?

Do you feel that other staff members have embraced THN (prompt – if not, why not? – can you tell me a bit more about this?)

So far as I understand, the invention to date has been delivered by *(identify most active individuals or groups – this response could potentially range from no-one to all relevant staff groups)*

- Is this impression correct?
- *(If relevant)* Why do you think these staff members have embraced THN?
- *(If relevant)* Why have others not embraced it?

*Listen for examples of perceived synergy or misalignment with beliefs, values, and priorities of specific individuals, certain staff groups/job roles, or organisation or a whole (e.g. this has ‘added’ to our response; the organisational focus is on emergency response, not prevention)*

- 3.How will the intervention be delivered?

Now turning more specifically to how the intervention works in practice:

- What barriers do you think there are to providing THN?

*(Allow conversation to flow unprompted. Barriers identified in previous literature include provider knowledge/confidence, timing and location of the intervention, competing demands, readiness of the intended recipient, provider/recipient attitude to THN)*

- What enablers are there?

*(Allow conversation to flow unprompted. Enablers identified in previous literature include staff training, opportunity to discuss safety with patients, opportunity to empower recipients)*

- Reflecting on your experience with the intervention so far, are there ways in which the staff training, THN intervention delivery and / or patient care could be improved?

*6 months*

- What would you say about these issues now? *(e.g. were these concerns/hopes realised? Are there new concerns/hopes?)*
- *(If relevant)* How has the intervention been received by patients?

• 4. How will you assess the effects of the intervention?

In this trial we are looking at measures of effectiveness and cost-effectiveness using routine data. However, we also know that the people delivering an intervention may have a “feel” for whether or not it is going well or you may have access to information that is not included in the trial. This can be useful for us to understand.

So, aside from waiting for the trial results, what indicators are there for you about the success or otherwise of the intervention?

*(Allow conversation to flow unprompted. Indicators could be present in patient records not available to the study, personal experience, anecdote, etc. The information could include awareness of reasons for missed opportunities to deliver, numbers of people refusing intervention, positive/negative response of different groups, etc)*

How does/might this this information affect your/your team’s practice in delivering the intervention?

• How have you assessed the effects of the intervention?

Feedback from staff last time we were here suggested that people felt *(summarise whether or not people felt trial/intervention initially going well and why they thought this)*

- Has anything changed since then? What has changed?
- *(If relevant)* What do you think has made this change come about? *(e.g. what factors have contributed to increase or decrease in THN being offered or accepted? How and why have internal processes been modified and with what effect?)*

As you know, this service has been offering THN as part of a feasibility trial

- Is this something you would like to see continue here at X service beyond the trial? Why/why not?
- What, if anything, would need to change/be put in place to make this as successful as possible?
- Is this intervention something you think should be expanded to other Emergency Department/ambulance services? Why/why not?
- What advice, if any, would you have about this? *(e.g. Should this be all EDs/services or targeted areas? What support/systems need to be in place?)*
- Thank you, that is all we planned to cover today. Before we finish, does anyone have anything further they wish to add?

Other questions to consider

Why have we found it difficult to recruit staff to the study? Is there anyone who you feel that we would benefit from speaking to? How do you think people feel about treating this population group
