# Supplementary material for: Is a randomised controlled trial of take home naloxone distributed in emergency settings likely to be feasible and acceptable? Findings from a UK qualitative study exploring perspectives of people who use opioids and emergency services staff
Source: BMC Emerg Med. 2024 Apr 29;24:75. doi: 10.1186/s12873-024-00987-y (PMC11057101; doi:10.1186/s12873-024-00987-y)
Supplement: Supplementary file 2 — Supplementary Material 2 [file 12873_2024_987_MOESM2_ESM.docx]

Topic guide - people who use opiates or family member interview or focus groups

**Introduction (and recap of consent and ground rules for focus groups)**

**Researcher script:** Thank you for [coming to this group today / taking part in this interview today]. The reason we have invited you is that we would like to hear your views on a new project about opioid overdose (that is, overdoses on heroin or opiate medications such as methadone, or fentanyl). During this project, people who are at risk of having an overdose may be given their own supply of a medication which reverses overdose.

[For focus groups: Before we start, I’d just like to recap on some key points from the participant information sheet you already have. Because there are a few of us here, I’d also like to suggest some “ground rules” to help the conversation run smoothly:

Before we begin, I would like to direct everyone’s attention to the consent form in order to remind ourselves that this group is voluntary, anyone can leave at any time, or opt not to answer specific questions, and there will be no consequences of doing so.

In giving consent to participate you have all agreed to keep what is discussed here confidential. This means not sharing other participant’s identities, or any information which might lead to someone being identified, outside of this room. We, the researchers, will not share personally identifying information such as your name outside this space. The only limit to this is if we were concerned you or at someone else is at risk of harm, in which case we’d need to get help in order to keep people safe.

Whilst inside the room we hope that everyone can be as supportive and respectful of one another as participants. It helps if only one person talks at a time. If you want to say something but are having trouble joining the conversation, please just raise your hand

We would like to extend our thanks to everyone for consenting to participate, and for giving us your time in being here today.]

- With your permission, we would like to tape record the conversation. This will later be typed up, and any personally identifying information such your names will be removed
- We are interested in your opinions and experiences and there are no right or wrong answers
- The topic of the conversation is potentially upsetting. Please don’t feel you have to take part today
- If you do take part, please just say if you would like to take a break at any point. If you decide you want to stop altogether, that is also absolutely fine
- The conversation will take up to 1.5 hours (up to 1 hour for interviews)
- We have some information about local services that we will give out at the end

Do you have any questions? Are we ok to start?

**Overdose experiences**

**Researcher script:** Just to begin, I’d like to get an idea of whether or not opioid overdose has been an issue for you or people around you.

- Briefly, what experiences of overdose have you had? *(Listen/prompt for: whether or not self/ family member/friend has overdosed, whether witnessed overdose, how frequent/rare, etc)*
- **If have experience of own or others’ overdose**
- What did you or others around you do when the overdose happened? [If person has multiple overdose experiences ask to focus on recent or typical example] *(Listen/prompt for: who was present, actions taken by self and others in response to overdose, mention of naloxone, outcomes)*
- **If have NO experience of own or others’ overdose**
- If you/a family member or friend overdosed, what do you think you and the people around you would do? *(Listen/prompt for: actions would and wouldn’t take, mention of naloxone)*

**Naloxone**

**Researcher script:** OK, thanks for sharing these experiences and thoughts about overdose. **[If naloxone hasn’t yet been mentioned also say** “There is a medication called Naloxone which can be given to a person who has overdosed to reverse the overdose. You may have already heard about this medication or it may be new to you”]

- What do you know about Naloxone? *(Responses to listen for/prompt include: how administered, perceptions/experiences of positive and negative effects, whether know temporary effect only, availability)*
- (If not already covered) Have you or someone you know ever received Naloxone to reverse an overdose? *(Listen/prompt for: how recently, where Naloxone from, who gave it, what were the outcomes, what would have happened without Naloxone)*

**Take Home Naloxone**

**Researcher script:** (If not already covered) There have been some programs in the UK and other countries to give people who are risk of overdose their own Naloxone. People are given a Take Home Naloxone “kit” to take away with them in case they or someone else has an overdose. People are also usually given some training in how to give the Naloxone and how to look after a person who has overdosed.

- What do you think would be the benefits giving out Take Home Naloxone? What would be the challenges? *(Listen/prompt for: preventing deaths, encourage drug use, discourage ambulance calls, need to carry/store, etc)*
- Who do you think should/shouldn’t be given Take Home Naloxone? Why do you think this? *(Listen/prompt for: families/friends of people at risk of an overdose)*

**Feasibility of distributing Take Home Naloxone via paramedics and ED staff**

**Researcher script:** In the UK, Take Home Naloxone has mainly been given out by drug services. However, it is not available in all areas, and many people who are at risk of overdose do not use drug services – so many people who could potentially benefit from having Take Home Naloxone do not have it. We are trying to see whether giving it out via **ambulance paramedics** & **hospital emergency departments** is a good idea.

At the moment, people in Hull/Bristol who have had an overdose (or who may be at risk of overdose) are being offered Take Home Naloxone by local paramedics and emergency department staff. So as well as making sure the person is ok, the staff are giving patients their own Naloxone "kit" to keep/take home. The kit includes a preloaded syringe of Naloxone which can be used on anyone who has overdosed.

This is the Take Home Naloxone kit (show and give brief overview of kit).

The hospital and ambulance staff are also teaching people how to use the kit and what else they can do to help keep someone who has overdosed alive. This training takes about 10 minutes and includes information about basic life support and calling emergency services.

- What, if anything, have you heard/do you know about Take Home Naloxone being given out by Bristol/Hull paramedics and Emergency Department?
- What are your thoughts about Take Home Naloxone being given out by Bristol/Hull:

(a) Paramedics (either in the hospital or in the community)?

(b) Emergency Department staff (in the hospital)?

*(Listen /prompt for: whether or not distribution in these settings is a good idea, suitability of each group to distribute, whether other settings/distributors preferred and why)*

- What could be the advantage of the paramedics and Emergency Department giving Take Home Naloxone? What could be the problems? Advantages/problems with the required 10 minute training? *(Listen /prompt for: whether timing right for patient, whether training likely to be possible)*
- Do you think you would take a kit if you were offered one? Why/why not? *(Listen /prompt for: barriers including kit size, storage/carrying of kit, time)*
- (If not already covered) Looking at this Take Home Naloxone kit:
- How willing would you be to use it on someone who had overdosed?
- How confident do you feel that you be able to use it on someone who had overdosed/that someone would be able to use it on you?
- What effect do you think having the Take Home Naloxone kit might have, *or has already had*, on you or other people’s behaviour:
  - willingness to call an ambulance? Try resuscitation?
  - staying with the person who has overdosed for a while? (as naloxone wears off after 20-30 minutes)
  - doses of opioids taken?

*(Listen /prompt for: whether able to recognise opioid overdose, comments on design of kit)*

**Final comments**

Thank you for your contribution today. Before we finish up, was there anything further you wanted to say?
